# The Role of Adopted Orphan Nuclear Receptors in the Regulation of an Organic Anion Transporting Polypeptide 1B1 (OATP1B1) under the Action of Sex Hormones

**DOI:** 10.3390/cimb45120600

**Published:** 2023-11-29

**Authors:** Aleksey V. Shchulkin, Yulia V. Abalenikhina, Aleksandr A. Slepnev, Egor D. Rokunov, Elena N. Yakusheva

**Affiliations:** Department of Pharmacology, Ryazan State Medical University, 390026 Ryazan, Russia; abalenihina88@mail.ru (Y.V.A.); a.slepnev@rzgmu.ru (A.A.S.); e.yakusheva@rzgmu.ru (E.N.Y.)

**Keywords:** organic anion transporting polypeptide 1B1, estradiol, testosterone, progesterone, adopted orphan receptors

## Abstract

Organic anion transporting polypeptide 1B1 (OATP1B1) is an influx transporter protein of the SLC superfamily, expressed mainly in the liver and some tumor cells. The mechanisms of its regulation are being actively studied. In the present study, the effect of sex hormones (estradiol, progesterone and testosterone) on OATP1B1 expression in HepG2 cells was examined. The role of adopted orphan receptors, farnasoid X receptor (FXR), constitutive androstane receptor (CAR), pregnane X receptor (PXR) and liver X receptor subtype alpha (LXR*a*), was also evaluated. Hormones were used in concentrations of 1, 10 and 100 μM, with incubation for 24 h. The protein expression of OATP1B1, FXR, CAR, PXR and LXR*a* was analyzed by Western blot. It was shown that estradiol (10 and 100 μM) increased the expression of OATP1B1, acting through CAR. Testosterone (1, 10 and 100 μM) increased the expression of OATP1B1, acting through FXR, PXR and LXR*a*. Progesterone (10 and 100 μM) decreased the expression of OATP1B1 (10 and 100 μM) and adopted orphan receptors are not involved in this process. The obtained results have important practical significance and determine ways for targeted regulation of the transporter, in particular in cancer.

## 1. Introduction

Organic anion transporting polypeptides (OATP) are members of the superfamily of proteins, encoded by the *SLCO* (Solute carrier organic anion) genes. The 11 human OATPs are classified into six families and subfamilies, based on the similarity of their amino acid sequences. OATPs are expressed in epithelial tissues throughout the human and animal body (mainly in the liver, kidneys, histo-hematic barriers) and transport mainly amphipathic molecules [1]. One of the most studied OATP transporters is OATP1B1 (OATP-C, OATP2, LST-1), which was first cloned in 1999 by three independent scientific groups [2,3,4].

OATP1B1 is considered to be a liver-specific transporter that is expressed on the basolateral membrane of hepatocytes and mediates the transport of substrates into cells (influx), where they will undergo biotransformation [4]. Also, OATP1B1 expression has been reported in cancer cells, for example, colon cancer tissue [5] and ovarian cancer tissue samples and cell lines (SK-OV-3) [6]. In regard to its transport functions in cancer cells, OATP1B1 is implicated to play the role in paclitaxel uptake in ovarian cancer cells [6].

The substrates of this transporter are various endogenous compounds, including bilirubin, 17-β-glucuronosyl estradiol, leukotriene C4 and estrone-3-sulfate [7], as well as a number of drugs, such as statins, antibiotics, angiotensin II receptor antagonists, angiotensin converting enzyme inhibitors, cytostatics, rifampicin and the natural toxins microcystin and phalloidin.

The mechanisms of the regulation of OATP1B1, as well as other OATP transporters, are being actively studied at the present [8]. For example, it has been shown that polymorphisms in the *SLCO1B1* gene, encoding OATP1B1, are usually associated with impaired transporter function and changes in its subcellular localization [9]. A number of OATP1B1 inhibitors have been described, for example, gemfibrozil, cyclosporine A, rifampicin, clarithromycin and erythromycin [10], which are capable to reduce the functional activity of the transporter due to the interaction with its molecule [11,12]. One of the main mechanisms of OATP1B1 regulation is the change in the expression of its gene (*SLCO1B1*), in particular under the action of transcription factors [13].

Orphan receptors are members of the nuclear receptor superfamily. They received their name due to the fact that, unlike classical nuclear receptors, endogenous ligands were not established for them [14]. At the moment, it is believed that the ligands of the farnasoid X receptor (FXR) are bile acids, of the constitutive androstane receptor (CAR)—androstan, of the pregnane X receptor (PXR)—pregnenolone 16*a*-carbonitrile and of the liver X receptor subtype alpha (LXR*a*)—oxysterols [15,16,17]. That is why these receptors were subsequently classified as “adopted” orphan nuclear receptors [17].

The important role of FXR in the regulation of bile acid synthesis, of CAR and PXR in the intracellular metabolism and redox balance and of LXR*a* in the lipid and cholesterol metabolism has been proved. In addition, all these receptors (FXR, CAR, PXR, LXR*a*) can participate in the regulation of enzymes of the I and II phases of biotransformation (for example, cytochrome P450 isoenzymes), as well as transporter proteins [18].

A number of studies have shown that sex hormones can affect the activity of transporter proteins, for example, P-glycoprotein [19,20], as well as the functioning of the FXR, CAR, PXR and LXR*a* receptors. It was shown that estradiol suppressed the expression of FXR through activation of ER*a* in HEK293T cells cotransfected with pcDNA3.1, FXR and the estrogen receptor α (ER*a*) together with the retinoid X receptor [21]. It was determined that in AML-12 cells, a differentiated untransformed mouse hepatocyte cell line, the testosterone metabolite androsterone, had directly activated FXR [22]. It was established that estradiol increased CAR expression, while testosterone and progesterone suppressed it in the human hepatocellular carcinoma HepG2 cell line, transfected by the mCAR expression plasmid, and showed high constitutive expression of mCAR with activation of the enhancer element NR1 [23]. It was found out that estradiol, progesterone and dihydrotestosterone had activated PXR in CV-1 cells, co-transfected with the tk(MH100)4-luc reporter gene and a chimeric receptor, consisting of the GAL4 DNA-binding domain and the PXR ligand binding domain [24]. It was found that the level of LXR*a* decreased significantly after ovariectomy in Sprague Dawley rats and the administration of estradiol increased the level of this receptor in the liver [25]. It has been shown that testosterone (1–10 nM, 24–72 h) increased gene expression and the amount of the LXR*a* protein in human monocytes differentiated into macrophages (THP-1 cell line) [26].

The influence of sex hormones, as well as gender features on the expression and functioning of OATP1B1, have not been practically studied. Therefore, the aim of this work was to study the effect of sex hormones (estradiol, progesterone and testosterone) on the expression of OATP1B1 and to assess the role of adopted orphan nuclear receptors (farnasoid X receptor, constitutive androstane receptor, pregnane X receptor, liver X receptor subtype alpha) in this process.

## 2. Materials and Methods

### 2.1. HepG2 Cell Line

The study was performed in a human hepatocellular carcinoma (HepG2) cell line (American Type Culture Collection, Manassas, VA, USA). These cells express all major transporters and transcription factors of human hepatocytes, including OATP1B1, CAR, PXR, FXR, LXR*a* [27,28]. The cells were cultured at 37 °C and 5% CO_2_ in Dulbecco’s modified Eagle’s medium containing 15% fetal bovine serum, 2 mM l-glutamine, 100 units/mL penicillin G and 100 μg/mL streptomycin (all components manufactured by Sigma-Aldrich, St. Louis, MO, USA).

### 2.2. The Study Design

The cells were cultured in 6-well plates. Estradiol (Sigma-Aldrich, USA), testosterone (Sigma-Aldrich, USA), progesterone (Sigma-Aldrich, USA) were added to the cell monolayer at concentrations of 1, 10 and 100 μM and incubated for 24 h. Control cells had been incubated for 24 h in the medium with the addition of ethanol (solvent of the tested substances) at the final concentration of 0.01%.

To test the role of adopted orphan receptors in the regulation of OATP1B1 by sex hormones, the receptors were blocked by specific inhibitors: the inhibitor of FXR—tauro-β-cholic acid at a concentration of 200 μM (β-TA, Sigma-Aldrich, USA) [29], the inhibitor of CAR—10 μM 5-[(Diethylamino)acetyl]-10,11-dihydro-5*H*-dibenzo[b,f]azepin-3-yl]ethyl ether of carbamic acid (CINPA 1, Tocris, Bristol, UK) [30], the inhibitor of PXR—10 μM ketoconazole (Sigma-Aldrich, USA) [31], the inhibitor LXR*a*—30 μM 3-(3,4-Dimethoxyphenyl)-*N*-[4-(trifluoromethyl)phenyl]-2-propenamide, *N*-(4-Trifluoromethylphenyl) 3,4-dimethoxycinnamide (TFCA, Sigma-Aldrich, USA) [32].

### 2.3. Western Blot

Protein expression of OATP1B1, FXR, CAR, PXR and LXR*a* in HepG2 cells was analyzed by Western blot.

To assess the expression of OATP1B1, cells were lysed with 100 µL of RIPA buffer (Sigma-Aldrich, USA) with protease inhibitors (Sigma-Aldrich, USA) per 10^7^ cells for 30 min at +4 °C and constant stirring. FXR, CAR, PXR and LXR*a* expression were tested in the nuclear fraction of the cells. For this purpose, cells were lysed with ReadyPrep Protein extraction kit (Cytoplasmic/Nuclear) (Bio-Rad, Hercules, CA, USA) with protease inhibitors (Sigma-Aldrich, USA).

Protein concentrations were quantified with Pierce Coomassie Plus (Bradford) Assay Kit (ThermoFisher, Waltham, MA, USA) [33]. The samples were mixed with Laemmli buffer (Bio-Rad, USA) containing 50 mM of β-mercaptoethanol (Bio-Rad, USA) in a 1:2 ratio and incubated for 10 min at 70 °C. Proteins (20 μg/sample) were separated using 7.5% SDS–PAGE at 100 V for 90 min. Proteins were transferred onto nitrocellulose membranes by semidry blotting system (Transblot, Bio-Rad, USA). Subsequently, the membranes were blocked for 1 h with TBS 1% Casein Blocker (Bio-Rad, USA) and then incubated overnight at 4 °C with primary antibodies (OATP2 Polyclonal Antibody, PA5-113548, Invitrogen, Waltham, MA, USA; MB67 CAR Monoclonal Antibody, Invitrogen, USA; MA5-31808 PXR Monoclonal Antibody 1D12G1, Invitrogen, USA; PAC042Hu01 Polyclonal Antibody to Farnesoid X Receptor, Cloud-Clone Corp., Wuhan, China; PAC044Hu01 Polyclonal Antibody to Liver X Receptor Alpha, Cloud-Clone Corp., China) at a concentration of 1:200 in the blocking solution. Primary antibodies for OATP2, FXR and LXR were visualized using secondary goat antibodies (Goat anti-Rabbit IgG (H+L) Cross-Adsorbed Secondary Antibody, HRP, Invitrogen, USA) in 1:4000 dilution. Primary antibodies for CAR, PXR were visualized using secondary rabbit antibodies (Rabbit-anti-Mouse IgG (H+L) Secondary Antibody, HRP, Invitrogen, USA) in 1:4000 dilution. The duration of incubation with secondary antibodies was 1 h at room temperature.

The protein content was measured relative to the level of the housekeeping protein GAPDH (primary antibodies: GAPDH Loading Control Monoclonal Antibody (GA1R), DyLight 68 (Invitrogen, USA) at a concentration of 1:400; secondary antibodies: secondary rabbit antibodies to primary GAPDH antibodies—Rabbit-anti-Mouse IgG (H+L) Secondary Antibody, HRP (Invitrogen, USA) in 1:4000 dilution). Chemiluminescence was recorded using ChemiDocXRS+ (Bio-Rad, USA). The intensity of the obtained bands was analyzed densitometrically using ImageLab 6.0.0 software (Bio-Rad, USA).

### 2.4. Data Analysis

Statistical analysis was conducted using GraphPad Prism version 8.1.2. Data are presented as mean (M) ± standard deviation (SD). Differences among groups were determined using analysis of variance (ANOVA), followed by Dunnet’s test. *p*-values of <0.05 were considered to be statistically significant.

## 3. Results

### 3.1. The Effect of Sex Hormones on the Expression of OATP1B1 in HepG2 Cells

**Figure 1:**
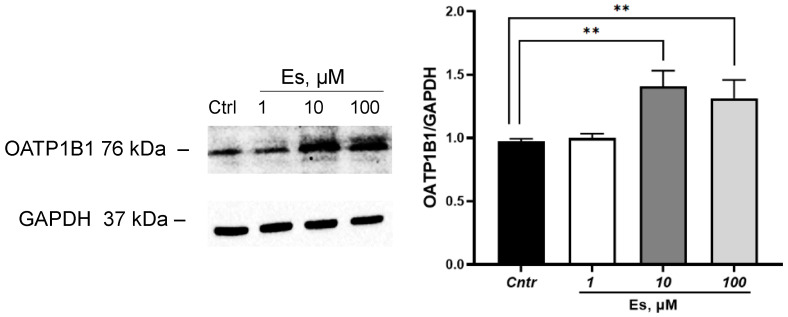
Effect of estradiol (1, 10, 100 µM, incubation for 24 h) on OATP1B1 expression in HepG2 cells. ** *p* < 0.01—statistically significant differences with the control, ANOVA, post hoc Dunnet’s test. Es—estradiol, Cntr—control.

Incubation of HepG2 cells with estradiol for 24 h at a concentration of 1 µM did not affect the expression of OATP1B1 and increased it at hormone concentrations of 10 and 100 µM by 40.8% (*p* = 0.002) and 31.3% (*p* = 0.007), respectively (Figure 1).

**Figure 2:**
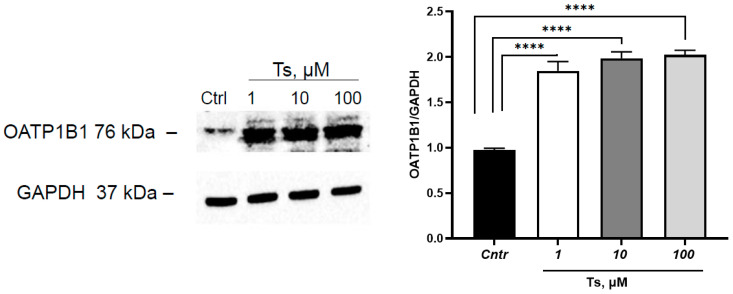
Effect of testosterone (1, 10, 100 µM, incubation for 24 h) on OATP1B1 expression in HepG2 cells. **** *p* < 0.001—statistically significant differences with the control, ANOVA, post hoc Dunnet’s test. Ts—testosterone, Cntr—control.

Testosterone at concentrations of 1, 10 and 100 µM increased the expression of OATP1B1 by 84.5%, 98.5% and 102.3%, respectively (*p* < 0.0001 for each group) (Figure 2).

**Figure 3:**
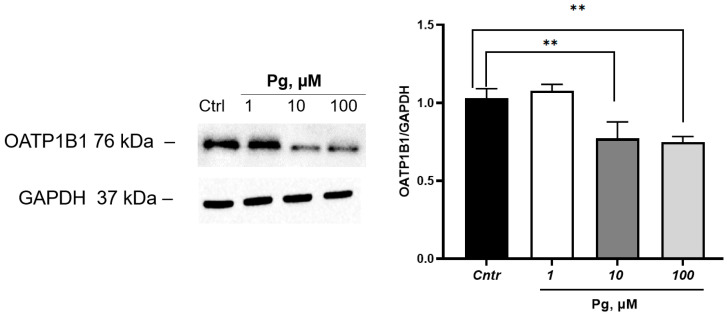
Effect of progesterone (1, 10, 100 µM, incubation for 24 h) on OATP1B1 expression in HepG2 cells. ** *p* < 0.01—statistically significant differences with the control, ANOVA, post hoc Dunnet’s test. Pg—progesterone, Cntr—control.

Progesterone at a concentration of 1 µM did not affect the expression of OATP1B1 but at concentrations of 10 and 100 µM decreased its expression by 24.9% (*p* = 0.003) and 27.2% (*p* = 0.002), respectively (Figure 3).

Thus, it was established that OATP1B1 is upregulated by estradiol and testosterone and downregulated by progesterone.

### 3.2. The Effect of Sex Hormones on the FXR, CAR, PXR, LXRa Levels

**Figure 4:**
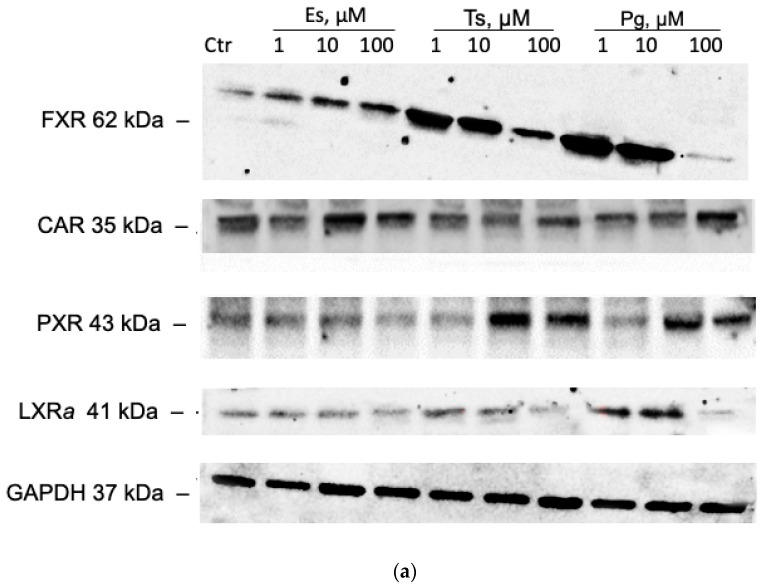

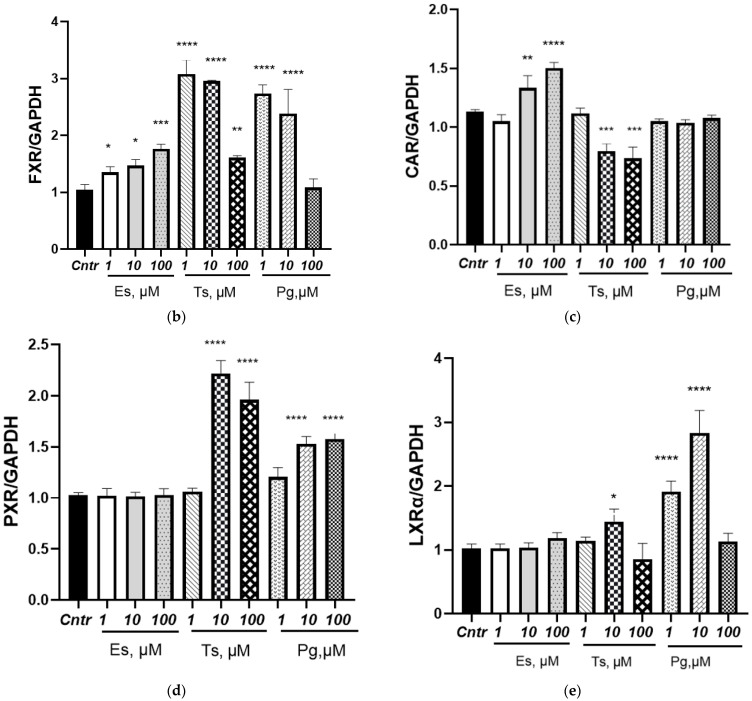
Effect of sex hormones (1, 10, 100 µM, incubation for 24 h) on the FXR, CAR, PXR and LXR*a* expression. (**a**) Results of Western blotting; (**b**) densitometric analysis of Western blotting of FRX; (**c**) densitometric analysis of Western blotting of CAR; (**d**) densitometric analysis of Western blotting of PXR; (**e**) densitometric analysis of Western blotting of LXR*a*. * *p* < 0.05; ** *p* < 0.01; *** *p* < 0.001; **** *p* < 0.0001—statistically significant differences with the control, ANOVA, post hoc Dunnet’s test. Es—estradiol, Ts—testosterone, Pg—progesterone, Cntr—control.

Estradiol at concentrations of 1, 10 and 100 µM increased the level of FXR by 26.2% (*p* = 0.05), 37.3% (*p* = 0.03) and 64.4% (*p* = 0.0007), respectively. Testosterone at concentrations of 1, 10 and 100 µM caused an increase in the level of FXR by 207% (*p* < 0.0001), 195.1% (*p* < 0.0001) and 60.7% (*p* = 0.008), respectively. Progesterone at concentrations of 1 and 10 µM increased the level of FXR by 162.5% (*p* < 0.0001) and 128.8% (*p* < 0.0001), respectively (Figure 4a,b).

Estradiol at concentrations of 10 and 100 µM increased the CAR level by 30% (*p* = 0.003) and 36.4% (*p* = 0.0001). Testosterone at a concentration of 1 µM did not affect the level of CAR but at concentrations of 10 and 100 µM decreased it by 31.9% (*p* = 0.0004) and 37.1% (*p* = 0.0002), respectively. Progesterone did not affect the expression of CAR in HepG2 cells (Figure 4a,c).

Estradiol at concentrations of 1, 10 and 100 µM did not affect the level of PXR in HepG2 cells. Testosterone increased the level of PXR by 116.3% and 88.5% (*p* < 0.0001 for both groups) at concentrations of 10 and 100 µM, respectively. Progesterone at concentrations of 10 and 100 µM increased the level of PXR by 53% (*p* < 0.0001) and 57.6% (*p* < 0.0001) (Figure 4a,d).

Estradiol did not affect the level of LXR*a* at all tested concentrations. Progesterone at concentrations of 1, 10 µM led to an increase in the level of LXR*a* by 75.2% (*p* < 0.00001) and 159.6% (*p* < 0.0001) (Figure 4a,c), while testosterone at a concentration of 10 µM increased the level of LXR*a* by 39.8% (*p* < 0.05).

Thus, since the level of receptors was assessed in the nuclear fraction of the cells, the results indicate that estradiol activates FXR and CAR, progesterone activates FXR, PXR, LXR*a*, and testosterone inhibits CAR and activates FXR, PXR, LXR*a*.

### 3.3. The Role of the Adopted Orphan Nuclear Receptors in the Induction of OATP1B1 under the Action of Estradiol

To test the role of the adopted orphan receptors in the regulation of OATP1B1 by sex hormones, we blocked receptors by specific inhibitors.

**Figure 5:**
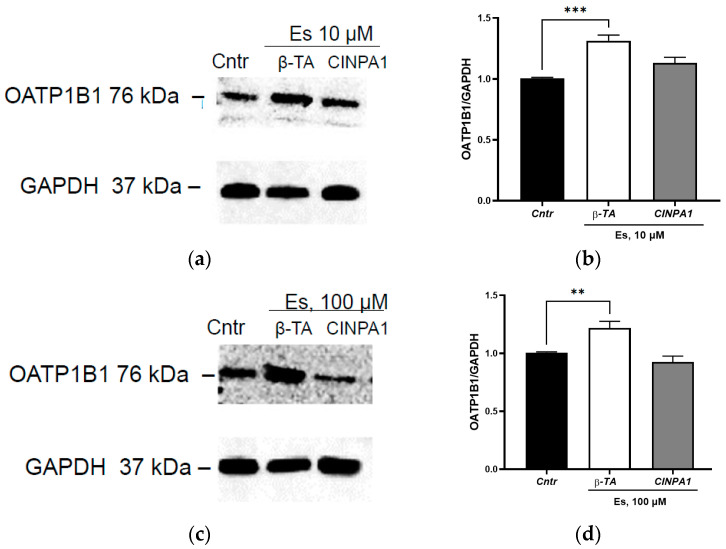
The role of the adopted orphan nuclear receptors in the induction of OATP1B1 under the action of estradiol at concentrations of 10 and 100 µM. (**a**) Results of Western blotting of OATP1B1 under the action of 10 µM estradiol + inhibitors of orphan receptors; (**b**) densitometric analysis of Western blotting of OATP1B1 under the action of 10 µM estradiol + inhibitors of orphan receptors; (**c**) results of Western blotting of OATP1B1 under the action of 100 µM estradiol + inhibitors of orphan receptors; (**d**) densitometric analysis of Western blotting of OATP1B1 under the action of 100 µM estradiol + inhibitors of orphan receptors. ** *p* < 0.01, *** *p* < 0.01—statistically significant differences with the control, ANOVA, post hoc Dunnet’s test. Es—estradiol, Cntr—control, β-TA—FXR inhibitor, CINPA1—CAR inhibitor.

The inhibition of FXR by β-TA did not prevent the induction of OATP1B1 under the action of estradiol. The expression of the transporter protein exceeded the control indicators by 31.7% (*p* = 0.009) when using the hormone at a concentration of 10 µM and by 28.1% (*p* = 0.003) when using estradiol at a concentration of 100 µM (Figure 5). At the same time, inhibition of CAR by CINPA1 prevented the induction of OATP1B1 under the action of estradiol at concentrations of 10 and 100 µM; its expression did not differ from the control (Figure 5). Thus, the effect of estradiol on OATP1B1 is mediated by transcription factor CAR.

Thus, apparently CAR is the only one to mediate the induction of OATP1B1 under the action of estradiol.

### 3.4. The Role of the Adopted Orphan Nuclear Receptors in the Induction of OATP1B1 under the Action of Testosterone

**Figure 6:**
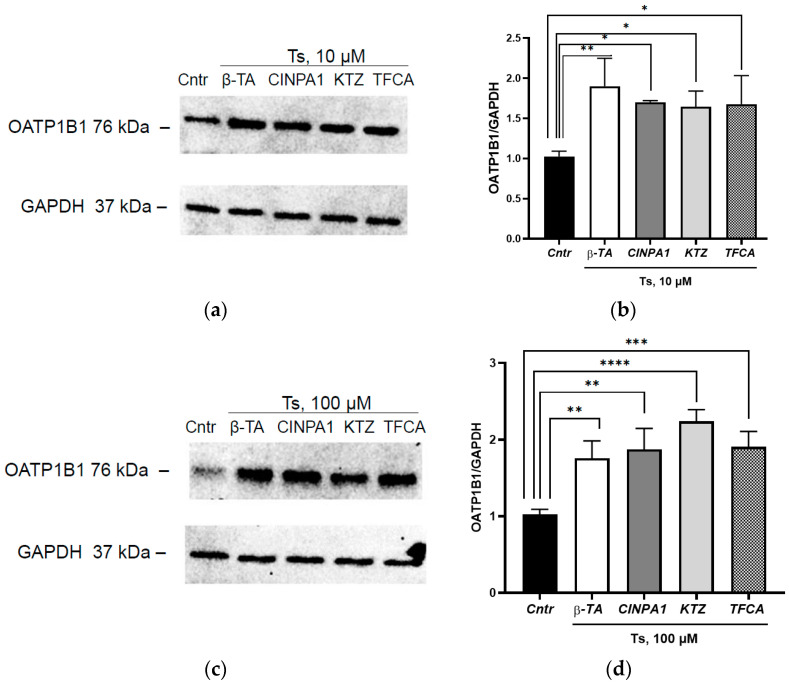
The role of orphan nuclear receptors in the induction of OATP1B1 under the action of 10 and 100 µM testosterone. (**a**) Results of Western blotting of OATP1B1 under the action of testosterone 10 µM + inhibitors of orphan receptors; (**b**) densitometric analysis of Western blotting of OATP1B1 under the action of 10 µM testosterone + inhibitors of orphan receptors; (**c**) Results of Western blotting of OATP1B1 under the action of 100 µM testosterone + inhibitors of orphan receptors; (**d**) densitometric analysis of Western blotting of OATP1B1 under the action of 100 µM testosterone + inhibitors of orphan receptors. * *p* < 0.05, ** *p* < 0.01, *** *p* < 0.001, **** *p* < 0.0001—statistically significant differences with the control, ANOVA, post hoc Dunnet’s test. Ts—testosterone, Cntr—control, β-TA—FXR inhibitor, CINPA1—CAR inhibitor, KTZ (ketoconazole)—PXR inhibitor, TFCA—LXR*a* inhibitor.

Isolated inhibition of FXR, CAR, PXR and LXR*a* had no effect on the inducing effect of testosterone on OATP1B1; the expression of the transporter protein exceeded the control indicators in all series by 85.2% (*p* = 0.004), 65.6% (*p* = 0.02), 60.2% (*p* = 0.03) and 63.4% (*p* = 0.02), respectively, when using testosterone at a concentration of 10 µM and by 71.1% (*p* = 0.004), 82.5% (*p* = 0.001), 118.0% (*p* < 0.0001) and 85.8% (*p* = 0.0009) at a testosterone concentration of 100 µM (Figure 6).

**Figure 7:**
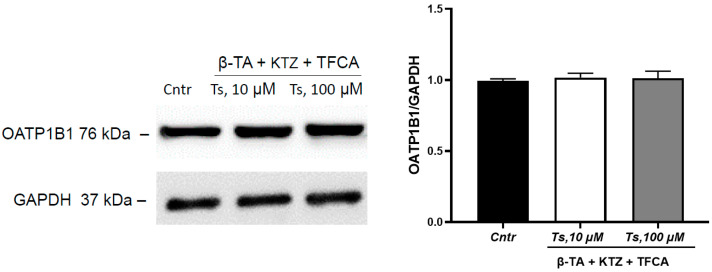
Effect of testosterone (10, 100 µM, incubation for 24 h) on OATP1B1 expression with simultaneous inhibition of FXR, PXR and LXR*a.* Ts—testosterone, Cntr—control, β-TA—FXR inhibitor, KTZ (ketoconazole)—PXR inhibitor, TFCA—LXR*a* inhibitor.

Taking into account the data obtained in the study that testosterone caused an increase in the amount of the PXR, LXR*a* and FRX in the nuclear fraction of the cells, an experiment with simultaneous inhibition of three receptors was performed. Simultaneous inhibition of FXR, PXR and LXR*a* prevented the increase in the expression of OATP1B1 under the action of testosterone at concentrations of 10 and 100 µM (Figure 7).

Thus, apparently all three receptors, FXR, PXR and LXR*a*, mediate the induction of OATP1B1 under the action of testosterone.

### 3.5. The Role of the Adopted Orphan Nuclear Receptors in the Inhibition of OATP1B1 under the Action of Progesterone

**Figure 8:**
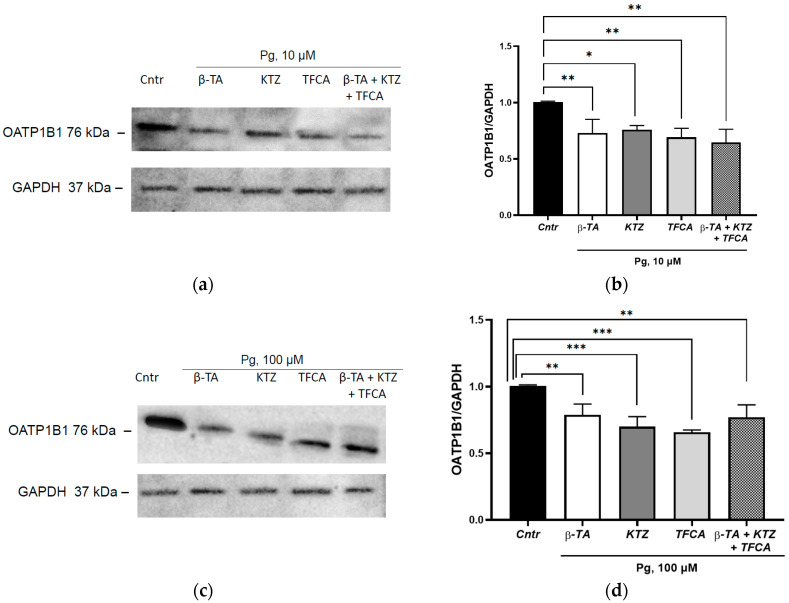
The role of orphan nuclear receptors in the inhibition of OATP1B1 under the action of 10 and 100 µM progesterone. (**a**) Results of Western blotting of OATP1B1 under the action of 10 µM progesterone + inhibitors of orphan receptors; (**b**) densitometric analysis of Western blotting of OATP1B1 under the action of 10 µM progesterone + inhibitors of orphan receptors; (**c**) results of Western blotting of OATP1B1 under the action of 100 µM progesterone + inhibitors of orphan receptors; (**d**) densitometric analysis of Western blotting of OATP1B1 under the action of 100 µM progesterone + inhibitors of orphan receptors. * *p* < 0.05, ** *p* < 0.01, *** *p* < 0.001, ANOVA, post hoc Dunnet’s test. Pg—progesterone, Cntr—control, β-TA—FXR inhibitor, KTZ (ketoconazole)—PXR inhibitor, TFCA—LXR*a* inhibitor.

Isolated inhibition of FXR, PXR and LXR*a* as well as simultaneous inhibition of the studied receptors had no effect on the inhibitory effect of progesterone on OATP1B1; the expression of the transporter protein decreased in all series by 27.1% (*p* = 0.004), 24.1% (*p* = 0.02), 30.6% (*p* = 0.03) and 35.4% (*p* = 0.02), respectively, when using progesterone at a concentration of 10 µM and by 21.2% (*p* = 0.0069), 30.5% (*p* = 0.0006), 34.3% (*p* = 0.002) and 22.9% (*p* = 0.0042) when using progesterone at a concentration of 100 µM (Figure 8).

The obtained results indicate that the studied receptors are not involved in the decrease in OATP1B1 expression under the influence of progesterone.

## 4. Discussion

The present study evaluated the effect of sex hormones (estradiol, progesterone and testosterone) on the expression of OATP1B1, as well as the role of adopted orphan receptors (PXR, CAR, FXR, LXR*a*) in the mechanisms of regulation of the transporter protein under these experimental conditions.

It was found that testosterone (1, 10 and 100 μM) and estradiol (10 and 100 μM) increase the expression of OATP1B1 in HepG2 cells, while progesterone (10 and 100 μM), on the contrary, reduces it.

The following mechanisms of OATP regulation have been described: changes in the expression of genes encoding transporters, epigenetic regulation, post-translational modification, phosphorylation and changes in the activity of the synthesized transporters (for example, inhibition of the transporter molecule) [13]. The main mechanism of regulation is the change in the expression of OATP under the influence of transcription factors; therefore, this mechanism was studied in this work. PXR, CAR, FXR and LXR*a* were chosen as the studied transcription factors since they are the main regulators of biotransformation enzymes and transporter proteins and also interact with sex hormones.

In our study, it was shown that estradiol increased the amount of FXR and CAR in the nuclear fraction of cells, testosterone increased the amount of FXR, PXR and LXR*a* and decreased the amount of CAR, and progesterone increased the level of FXR, PXR and LXR*a*. Since we analyzed the level of receptors in the nuclear fraction of cells, the obtained results indicate the activation of these receptors. To test the role of the adopted orphan receptors in the regulation of OATP1B1 by sex hormones, we blocked receptors by specific inhibitors:

Tauro-β-cholic acid was used as an FXR inhibitor. It is assumed that it interacts with the ligand-binding pocket of FXR and prevents the realization of its activity [29]. CINPA1 (CAR inhibitor not PXR activator 1) was used as a CAR inhibitor. It blocks the ligand binding domain of CAR and also suppresses its interaction with coactivators [30]. The inhibition of PXR was carried out using ketoconazole. Ketoconazole (an antifungal drug of the azole group) binds to the AF-2 region of the PXR ligand-binding domain and thus suppresses its activation [34]. N-(4-trifluoromethylphenyl) 3,4-dimethoxycinnamide (TFCA) was used to inactivate LXR*a*. It is believed that TFCA inhibits the activation of the ligand-binding domain of LXR*a* due to hydrogen bonding with Arg305 in the H5 region of this domain [32].

Isolated inhibition of FXR, CAR, PXR and LXR*a* had no effect on the inducing effect of testosterone on OATP1B1; however, simultaneous inhibition of PXR, FXR and LXR*a* prevented an increase in the expression of OATP1B1 under the action of testosterone. Thus, apparently three receptors, PXR, FXR and LXR*a*, mediate the induction of OATP1B1 under the action of testosterone. The results obtained are consistent with other works. Thus, the regions of the SLCO1B1 gene promoter from −3040 bp to −4070 bp or from −1480 bp to −2500 bp are assumed to be responsible for FXR binding, and the DR4 DNA hexamer motif localized in DR4-1 from +22 to +37 and DR4-2 from +32 to +47 is responsible for binding LXR*a* [35]. In human hepatocytes, it was shown that OATP1B1 is induced by rifampicin (a PXR inducer) [36]. Thus, all three transcription factors can play a role in the induction of OATP1B1 by testosterone.

Inhibition of FXR did not affect the induction of OATP1B1 under the action of estradiol; while inhibition of CAR prevented the induction of OATP1B1, its expression did not differ from the control. Thus, apparently only CAR mediates the induction of OATP1B1 by estradiol.

Isolated inhibition of FXR, PXR and LXR*a*, as well as simultaneous inhibition of all studied receptors, had no effect on the inhibitory effect of progesterone on OATP1B1; the expression of the transporter protein decreased in all series by progesterone. Thus, apparently progesterone implements its effect on OATP1B1 through other signaling pathways. On the other hand, in C57BL6 mice, it was shown that the 3ß-sulfated metabolite of progesterone epiallopregnanolone sulfate prevented the induction of FXR caused by cholic acid, which indicates the inhibition of FXR under the action of the metabolite of progestogen [37]. That is, a decrease in the amount of OATP1B1 may be associated with the inhibition of FXR by progesterone metabolites.

The expression of OATP1B1, like other influx transporters, in tumor cells promotes the penetration of cytostatic substrates into the cells and can increase the effectiveness of antitumor therapy [38,39]. Thus, the obtained data on the increase in the level of OATP1B1 by estradiol and testosterone can be useful in the complex therapy of cancer diseases in some cases.

FXR plays an important role in bile acid homeostasis. FXR and its hepatic and intestinal target genes transcriptionally regulate bile acid synthesis, detoxification, secretion and absorption in the enterohepatic circulation [40]. The obtained data on the participation of FXR in the up-regulation of OATP1B1 (bile acids are substrates of OATP1B1) by testosterone expand the existing understanding of the role of this receptor in the homeostasis of bile acid.

In similar studies, sex differences and the effect of sex hormones on the expression of transporters, members of the OATP superfamily, were revealed. For example, it has been shown that the expression of OATP1 in the kidneys is stimulated by testosterone but weakly inhibited by estrogen. This explains the gender differences in urinary excretion of glucuronidated steroids as OATP1 substrates [41,42]. The liver of male mice is dominated by Oatp1a1, and the expression of Oatp1a4 is higher in females. Oatp1a1 and Oatp3a1 predominate in the kidneys of females [43,44]. The expression of SLC3A1 genes in the liver is 2.35 times, and SLC10A1 is 1.48 times, higher in women, and SLC13A1 is 1.57 times higher in men [45].

## 5. Conclusions

Thus, in this study on HepG2 cells, it was found that: (1) testosterone increases the expression of the protein transporter OATP1B1, acting through FXR, PXR and LXR*a*; (2) estradiol increases the amount of the OATP1B1 transporter protein acting through CAR; (3) progesterone reduces the expression of the OATP1B1 transporter protein and its effect is not associated with the effect of the adopted orphan receptors CAR, PXR, FXR and LXR*a*. The obtained results have important practical significance and determine the ways of targeted regulation of the transporter, in particular, in cancer.

### The Study Limitations

The study limitations include the performance experiments on a single HepG2 cell line, as well as the lack of assessment of the role of sex hormone receptors (estrogen, progesterone and androgen receptors) in the regulation of OATP1B1.

## Data Availability

The data presented in this study are available on reasonable request from the corresponding author.
